# A Case of Acute Catatonia Precipitated by Psychosis Successfully Treated With Lorazepam: A Case Report

**DOI:** 10.7759/cureus.49648

**Published:** 2023-11-29

**Authors:** Ateeba Ahmed, Ragini Patil, Imyarila Longkumer

**Affiliations:** 1 Psychiatry, Jawaharlal Nehru Medical College, Datta Meghe Institute of Higher Education and Research, Wardha, IND

**Keywords:** tertiary care hospital, schizoaffective disorder, neuropsychiatric syndrome, lorazepam, psychosis, catatonia

## Abstract

Catatonia is a multifaceted neuropsychiatric syndrome characterized by a spectrum of psychomotor disturbances that can severely impact the well-being of affected individuals. It may manifest as a primary psychiatric disorder or be associated with underlying medical, neurological, or psychiatric conditions. This case report details the clinical journey of a 22-year-old male who initially presented with psychotic symptoms and subsequently developed acute catatonia within three days of admission to a tertiary care hospital. The patient was successfully treated with intravenous lorazepam, resulting in a rapid and complete resolution of his catatonic state. This case underscores the intricate relationship between psychosis and catatonia and highlights the efficacy of lorazepam in managing catatonia. Recognition and timely intervention are pivotal for optimal patient outcomes. By shedding light on the importance of early diagnosis, comprehensive evaluation, and targeted treatment for catatonia, this case report adds to the body of knowledge in psychiatric practice. It underscores the need for clinicians to consider catatonia as a potentially reversible condition, particularly in individuals with psychotic disorders, and emphasizes the critical role of lorazepam in its management.

## Introduction

Catatonia is a complex neuropsychiatric syndrome characterized by a range of psychomotor disturbances, including mutism, negativism, motor abnormalities, and other behavioral anomalies [1]. Around 90,000 cases of catatonia occur in the United States per year. Catatonia has a reported prevalence of 7% to 38% in psychiatric patients [2]. People with mood disorders, psychosis, increased age, increased frequency of depressive episodes, and cognitive impairment are at an increased risk for developing catatonia [3,4]. It can manifest as a primary psychiatric condition or as a feature of various underlying medical, neurological, or psychiatric disorders. Catatonia is often a medical emergency; prompt recognition and intervention are essential for favorable outcomes [5].

This case report presents a compelling illustration of the interplay between psychosis and catatonia, emphasizing the rapid and successful management of catatonia using intravenous lorazepam. The report underscores the significance of early diagnosis, proper evaluation, and targeted treatment for catatonia, and the necessity for a comprehensive understanding of this neuropsychiatric syndrome. Furthermore, it accentuates the importance of considering catatonia as a reversible condition that can significantly affect the clinical course of patients with psychotic disorders [6]. This case report contributes to the growing body of literature on the recognition and management of catatonia in psychiatric practice.

## Case presentation

A 22-year-old male presented with a chief complaint of auditory hallucinations, paranoid delusions, and disorganized speech. These symptoms had been gradually escalating over two weeks. Family members reported that he had been exhibiting bizarre behavior, social withdrawal, and deteriorating personal hygiene during this period. The patient's presenting symptoms were characterized by auditory hallucinations, where he reported hearing voices that were not externally present, as well as paranoid delusions, leading him to hold false and fixed beliefs. Additionally, he exhibited disorganized thought processes, marked by incoherent speech. These symptoms were a source of significant distress for the patient.

Upon the initial evaluation in the outpatient department, the patient exhibited prominent symptoms of psychosis, including auditory hallucinations and disorganized thought processes. Recognizing the severity of his condition and its substantial impact on daily functioning, a decision was made to admit him to the psychiatric ward for a more comprehensive assessment and management. The preliminary diagnosis centered around a primary psychotic disorder.

The patient's clinical presentation underwent a notable evolution during the first three days following admission to the psychiatric ward. His condition deteriorated significantly, marked by a progressive muteness and a pronounced reduction in motor activity. Additionally, he exhibited characteristic features of catatonia, such as waxy flexibility, negativism (an active resistance to following commands or responding to external stimuli), and purposeless agitation. In response to this concerning development, a detailed management plan was implemented to address the emergent catatonic features involving the administration of intravenous lorazepam. This intervention resulted in a rapid and substantial improvement in the patient's psychomotor symptoms, culminating in the complete resolution of the catatonic state. This strategic approach not only effectively managed the acute manifestation of catatonia but also facilitated subsequent psychiatric evaluations to refine the overall treatment plan, leading to a more targeted and comprehensive approach to the patient's underlying psychotic disorder.

Extensive diagnostic procedures were initiated to uncover potential etiological factors contributing to the patient's clinical presentation. Firstly, a comprehensive neurological examination was conducted, encompassing a thorough evaluation for any focal neurological deficits or unusual findings, all of which yielded no significant abnormalities. Additionally, laboratory investigations included routine blood tests such as a complete blood count, electrolyte level measurement, liver and kidney function assessment, and toxicology screening. Notably, all the results from these laboratory tests fell within the expected normal ranges.

In light of the escalating clinical symptoms and strong suspicion of catatonia, the decision was made to administer intravenous lorazepam. The initial dose of 2 mg was given, followed by 1 mg every 10 minutes until 6 mg was administered. Remarkably, within 30 minutes of the first dose of lorazepam, there was a noticeable improvement in the patient's psychomotor symptoms, with a complete resolution of catatonic features. The patient's underlying psychotic symptoms persisted, and a treatment plan involving antipsychotic medication was initiated for further management.

Over the following days, the patient continued to make progress, with the complete resolution of his catatonic symptoms. He became more responsive, engaged in communication, and displayed a general improvement in psychomotor activity. While the psychotic symptoms persisted, a comprehensive psychiatric evaluation eventually led to a diagnosis of schizoaffective disorder, and the patient was discharged with a treatment plan that included antipsychotic medication and scheduled outpatient follow-up.

## Discussion

The presented case underscores the intricate relationship between psychosis and catatonia and highlights the effective management of catatonia with intravenous lorazepam. The co-occurrence of catatonia and psychosis is well-documented in the literature [7]. Catatonia can be associated with various psychiatric conditions, including schizophrenia and mood disorders, and may emerge as a prominent clinical feature. This association has been reported in numerous studies [7,8]. In our case, the patient initially presented with psychotic symptoms, including auditory hallucinations and paranoid delusions, which later progressed to full-blown catatonia within three days. Such a rapid transition underscores the dynamic nature of neuropsychiatric symptomatology and the need for vigilant clinical assessment.

The efficacy of lorazepam in the management of catatonia is well-established. Lorazepam, a benzodiazepine with GABAergic mechanisms, is considered a first-line treatment for catatonia [6]. It acts by enhancing inhibitory neurotransmission, resulting in the rapid alleviation of catatonic symptoms [9]. In our case, the administration of intravenous lorazepam led to a swift and complete resolution of catatonia, further supporting its use as an effective treatment option.

Prompt recognition and intervention in catatonia are crucial. Delayed or misdiagnosed catatonia can lead to prolonged suffering and complications. Therefore, clinicians must maintain a high index of suspicion for catatonia, particularly when patients with psychotic disorders exhibit rapid deterioration in psychomotor function [10]. Furthermore, comprehensive psychiatric evaluations are essential to determine the underlying diagnosis, as demonstrated in our case, where a diagnosis of schizoaffective disorder was eventually established.

## Conclusions

In conclusion, while acknowledging the established use of lorazepam in managing catatonia, this case report aims to highlight a distinctive aspect regarding the rapidity of recovery observed. The emphasis here lies in the prompt and complete resolution of catatonic features within 30 minutes of administering lorazepam, a phenomenon that, to our knowledge, is not extensively documented in the existing literature. While lorazepam's efficacy in catatonia is well-established, the exceptional speed of recovery observed in this case prompts further consideration. The unique aspect of our report lies in the swift and comprehensive response to lorazepam, suggesting the potential for an unusually rapid resolution of catatonic symptoms. While rapid responses to lorazepam have been reported previously, the extent and immediacy of recovery observed in this case may warrant additional investigation. This presentation underscores the importance of early diagnosis and intervention in catatonia, particularly in the context of psychosis. It prompts clinicians to not only consider lorazepam as a standard treatment modality but also recognize that the speed of recovery may vary among individuals. While acknowledging the need for further research to substantiate these observations, this case report catalyzes raising awareness regarding the nuanced responsiveness of catatonia to lorazepam, potentially shedding light on variations in treatment outcomes within this patient population.
